# Progressive Multifocal Leukoencephalopathy Mimicking a Cerebral Vasculitis Flare

**DOI:** 10.7759/cureus.41094

**Published:** 2023-06-28

**Authors:** Sharafath Hussain Zahir Hussain, Kehinde O Sunmboye

**Affiliations:** 1 Medicine, University Hospitals of Leicester NHS Trust, Leicester, GBR; 2 Rheumatology, University Hospitals of Leicester NHS Trust, Leicester, GBR

**Keywords:** biologic treatment, jc polyomavirus, immunosupression, stroke, cerebral vasculitis, hiv-negative progressive multifocal leukoencephalopathy

## Abstract

We report a case of a 68-year-old woman with a background of primary cerebral vasculitis, which was diagnosed two years ago. She appeared to have had a recurrence of her symptoms with new onset history of expressive dysphasia, right-sided upper limb weakness, and right-sided facial weakness during a rheumatology clinic visit. The patient was on maintenance azathioprine for her cerebral vasculitis at the time of presentation. She had received a total of 2 g of rituximab through intravenous infusion, with a two-week interval between doses. Additionally, she had undergone intravenous cyclophosphamide treatment (15 mg/kg) following the standard vasculitis regimen for induction remission therapy, which was administered at the time of her diagnosis two years prior. Initial imaging on non-contrast computed tomography head after admission to the emergency department did not show any acute neurological findings. Further imaging studies revealed changes in the right parietotemporal white matter T2 hyperintensity with similar changes on the left frontal and left parietal lobes suggestive of progressive multifocal leukoencephalopathy (PML). A magnetic resonance imaging (MRI) of the brain conducted three months prior was found to be unremarkable. Cerebrospinal fluid (CSF) polymerase chain reaction (PCR) testing confirmed the presence of polyoma John Cunningham (JC) virus deoxyribonucleic acid (DNA). This case highlights that PML should be an important differential to consider in any immunocompromised patient who presents with new stroke-like features.

## Introduction

Progressive multifocal leukoencephalopathy (PML) is a rare, life-threatening demyelinating central nervous system disease caused by the reactivation of John Cunningham (JC) polyomavirus in immunocompromised individuals [1]. The JC virus is a ubiquitous human polyomavirus that establishes a latent infection in a majority of the population. However, in situations of compromised immune function, such as in patients receiving immunosuppressive therapies or those with underlying conditions that can affect the immune system, the virus can reactivate and lead to the development of PML [2]. It is characterized by the destruction of white matter in the brain, leading to a variety of neurological symptoms, and it has a poor prognosis [3]. The diagnosis of PML relies on several key factors, including clinical manifestations, characteristic neuroradiological findings in brain magnetic resonance imaging (MRI), and the presence of the JC virus in cerebrospinal fluid (CSF) [4]. Here, we report a case of PML in a patient with primary cerebral vasculitis.

## Case presentation

A 68-year-old woman with a history of primary cerebral vasculitis was undergoing maintenance treatment with azathioprine for her cerebral vasculitis condition. However, she had received a total of 2 g of rituximab through intravenous (IV) infusion, with a two-week interval between doses. Additionally, she had undergone IV cyclophosphamide treatment (15 mg/kg), which was administered using the standard vasculitis regimen for induction remission therapy at the time of her diagnosis two years earlier. She presented to the rheumatology clinic with a three-week history of weakness in the right upper limb, right-sided facial weakness, and expressive dysphasia. She was not on maintenance rituximab at the time of presentation as the drug was only used for induction remission therapy. She was currently only being treated with maintenance azathioprine 100 mg, orally, once a day, and low-dose prednisolone at 5 mg daily. She had a significant past medical history of previous lung adenocarcinoma three years ago, which was successfully treated through a left lower lobectomy and uniportal video-assisted thoracic surgery (VATS). Additionally, she underwent chemoprophylaxis two years ago to address latent tuberculosis treatment. She also had mixed cryoglobulinemia with systemic involvement, which responded well to a course of IV immunoglobulins three years ago.

**Figure 1:**
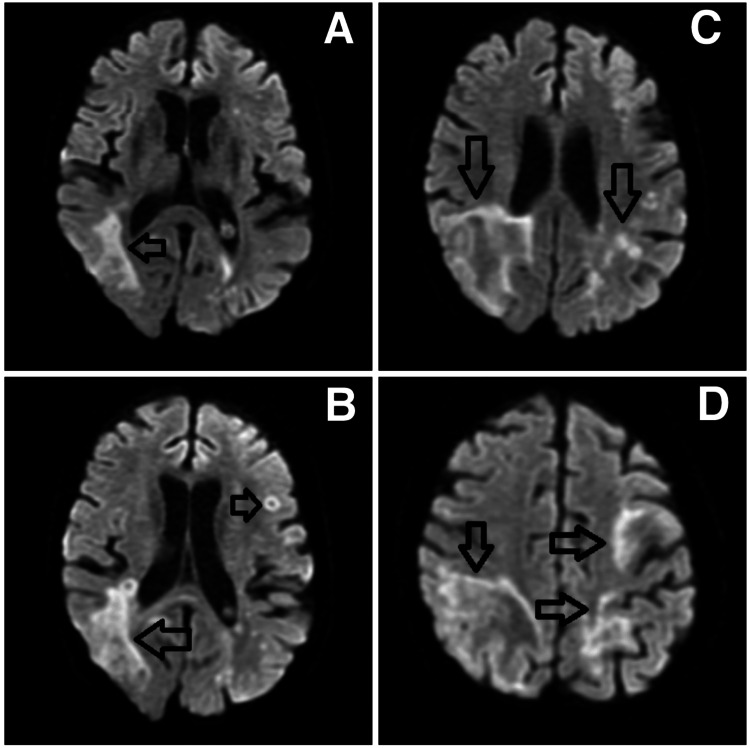
(A-D) Magnetic resonance imaging of the brain showing right parietotemporal white matter T2 hyperintensity (DWI sequence) and suggesting an inflammatory/demyelinating process. The relative lack of mass effect and DWI leading edge suggest progressive multifocal leukoencephalopathy. There are similar changes on the left in the frontal and parietal lobes. There is a mass effect associated with the white matter change, but it is modest. There is a leading edge of diffusion restriction associated with the hemispheric white matter change and some definite rings of restriction. DWI, diffusion-weighted imaging

For her current clinical presentation, she had been referred to the stroke unit for further evaluation. Upon admission, an initial non-contrast computed tomography was conducted, which did not reveal any acute pathology. Blood tests conducted showed an elevated C-reactive protein (CRP) level of 78 mg/dL and a decreased white cell count of 3.0 × 10^9 ^per liter. Other blood investigations yielded normal results. One week after admission, a subsequent MRI of the brain imaging (Figures 1A-1D) revealed white matter T2 hyperintensity, particularly in the right parietotemporal lobe as well as the left frontal and left parietal lobes. These findings were accompanied by a relative lack of mass effect and leading edge restriction on diffusion-weighted imaging (DWI). Following this, two days later, she had a lumbar puncture, and CSF polymerase chain reaction (PCR) detected 67,000 copies per milliliter of JC virus deoxyribonucleic acid (DNA). CSF profile, besides the detection of the JC virus, was normal. Tests for human immunodeficiency viruses (HIV), hepatitis B, and hepatitis C were also negative.

The patient’s hospital stay lasted for 20 days. During this time, she continued to deteriorate cognitively becoming noncommunicative and significantly disorientated. Extensive conversations were held with her family regarding her progressive cognitive decline. The case was discussed with a multispecialty multidisciplinary team (MDT), and it was collectively decided to refer her to palliative care input. Following discussions with the rheumatology team and the infectious diseases team, the decision was made to discontinue the administration of azathioprine for the patient. In preparation for her palliative care, anticipatory medications were prescribed as needed. Subsequently, she was discharged with a referral to community palliative care nurses to ensure continuity of support and management. Due to her poor prognosis, she died a few months after discharge as a result of PML.

## Discussion

PML caused by the JC virus is a destructive infection of oligodendrocytes [5]. The virus remains asymptomatic during primary infection, but severe deficiency of cellular immunity, particularly T-cell immunity, is necessary for reactivation [2,6]. 

In addition to HIV, hematological malignancies, and organ transplant recipients, PML has been recently observed in individuals treated with monoclonal antibodies such as natalizumab, rituximab, and efalizumab [6]. Rituximab has been linked to case series of 57 HIV seronegative PML cases [3]. The case fatality in these cases was 90%. PML diagnosed within the three months of the last rituximab dose had a case fatality rate of 100% and those diagnosed more than three months later had an 84% fatality rate. The presentation of these cases included confusion, hemiparesis, poor motor coordination, speech changes, and visual changes [3]. A median of six rituximab doses preceded PML diagnosis in this literature [3].

Our case report focuses on a patient with primary cerebral vasculitis who developed PML. In our case, the occurrence of PML was not considered to be directly linked to her previous rituximab infusion, which was administered two years before the onset of PML. Our assessment is based on existing literature, which suggests that the median onset time from the last rituximab dose to the development of PML is approximately 5.5 months and a median of six rituximab doses preceded PML diagnosis [3]. An MRI brain she had before the presentation as part of her routine monitoring for cerebral vasculitis did not show any features of PML. She was only on azathioprine and low-dose steroids at her index presentation. 

When presented with patients exhibiting stroke-like symptoms, it can be challenging to determine the underlying cause, particularly in the context of a history of primary cerebral vasculitis. PML can manifest in a manner that closely mimics a stroke, adding to the diagnostic complexity. Confusion in a patient with primary cerebral vasculitis can be attributed to either a new flare of the existing primary cerebral vasculitis, a new acute infection, or drug-related causes common in the elderly. Reflecting on our case, a high index of suspicion was required to look for a diagnosis of PML in a patient with stroke-like features who was immunocompromised with confusion and expressive dysphasia. A comprehensive evaluation that included a detailed clinical history, imaging studies, and relevant laboratory investigations was conducted, which was helpful to arrive at the diagnosis.

Our case report highlights the importance of screening for PML in autoimmune individuals receiving immunosuppressive therapy, who present with signs and symptoms of a stroke with confusion, even if the initial cranial imaging findings are normal. A review of the literature showed additional case reports, which highlight the potential association of PML in individuals with autoimmune diseases and immunosuppression, including an 80-year-old woman with antineutrophil cytoplasmic antibodies (ANCA)-associated renal vasculitis and a 27-year-old with systemic lupus erythematosus (SLE)-rheumatoid arthritis overlap syndrome [7,8].

Current treatment options for PML are limited. Immune reconstitution has been explored, but this can lead to PML immune reconstitution syndrome (PML-IRIS), particularly in HIV, multiple sclerosis, and natalizumab-associated PML. Maraviroc has been hypothesized to reduce the severity of PML-IRIS, but its clinical effectiveness has not been established [9]. Current research is exploring multiple drugs, including a promising compound called Oxindole GW-5074, which is still under trial [10]. 

## Conclusions

In this era of extensive biologics use, clinicians should be aware of an alternate diagnosis of PML in individuals with autoimmune diseases who are immunosuppressed and who present with stroke-like features with normal imaging initially. Although current treatment options are limited, early diagnosis of PML is important so that unnecessary investigations can be avoided and appropriate palliation and support can be offered to these patients as well as their families.
